# Supplementary figures and images for: A Vibrio cholerae viral satellite maximizes its spread and inhibits phage by remodeling hijacked phage coat proteins into small capsids
Source: eLife. 2024 Jan 11;12:RP87611. doi: 10.7554/eLife.87611 (PMC10945586; doi:10.7554/eLife.87611)

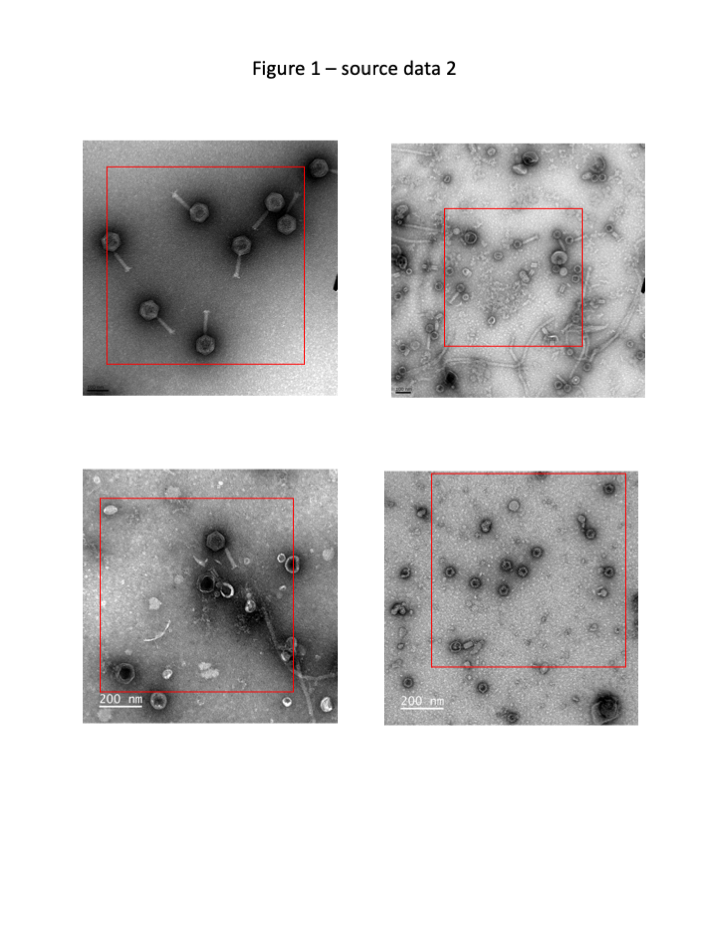

Supplement: Figure 1—source data 2. [file elife-87611-fig1-data2.zip › Figure_1_source_data_2_crop.tiff]

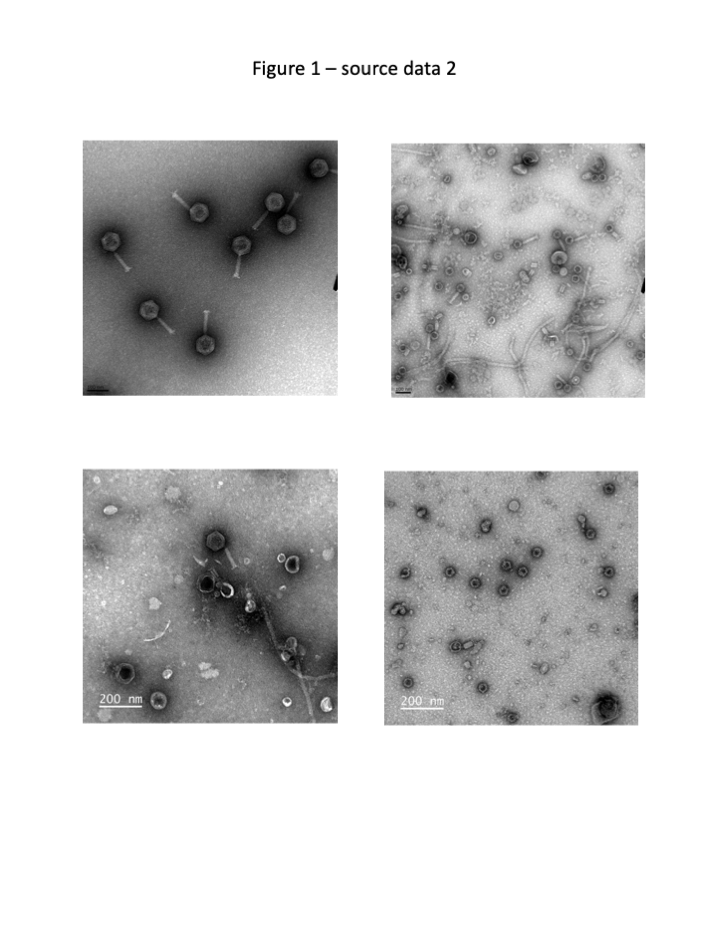

Supplement: Figure 1—source data 2. [file elife-87611-fig1-data2.zip › Figure_1_source_data_2.tiff]

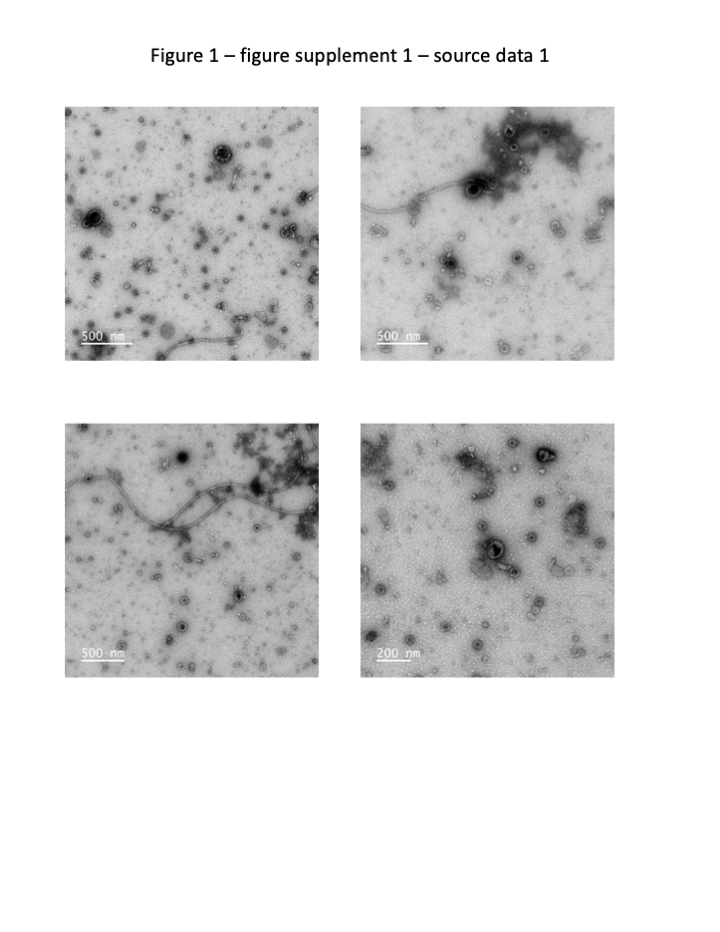

Supplement: Figure 1—figure supplement 1—source data 1. [file elife-87611-fig1-figsupp1-data1.zip › Figure_1_figure_supplement_1_source_data_1.tiff]

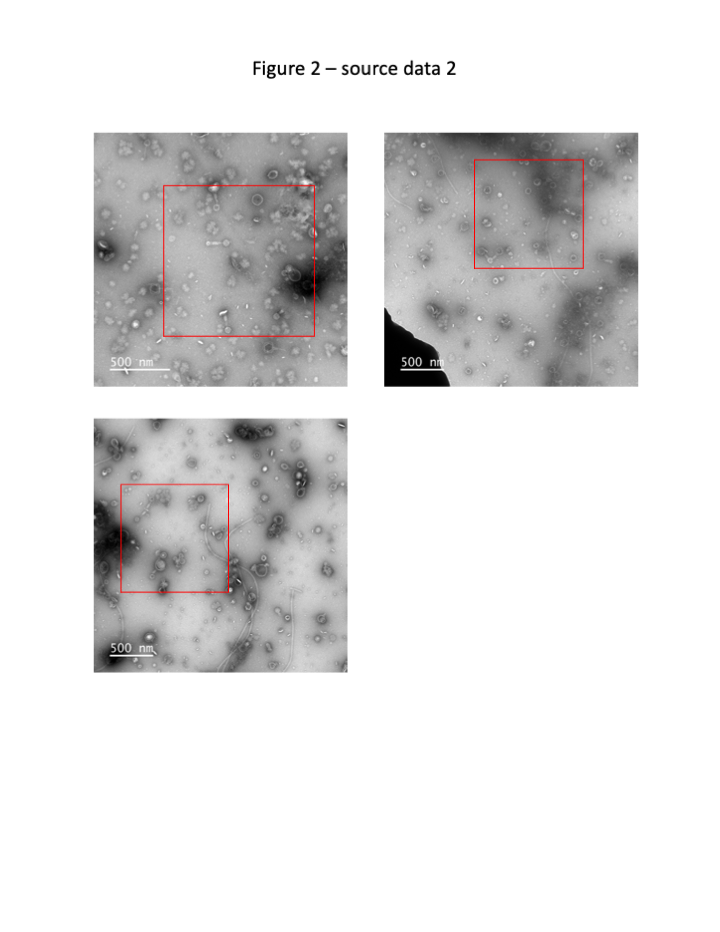

Supplement: Figure 2—source data 2. [file elife-87611-fig2-data2.zip › Figure_2_source_data_2_crop.tiff]

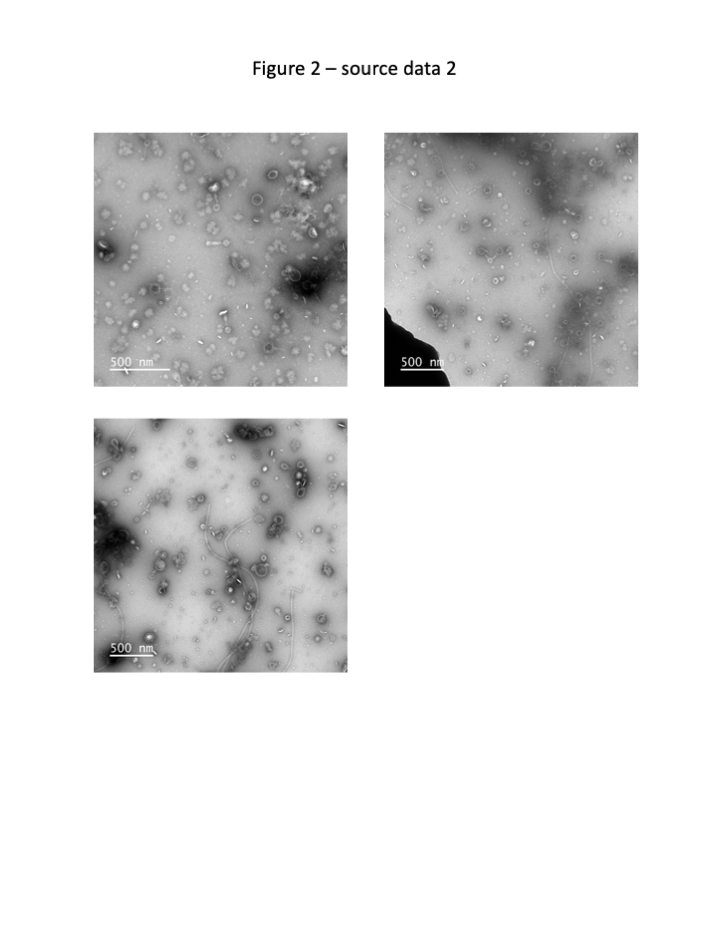

Supplement: Figure 2—source data 2. [file elife-87611-fig2-data2.zip › Figure_2_source_data_2.tiff]

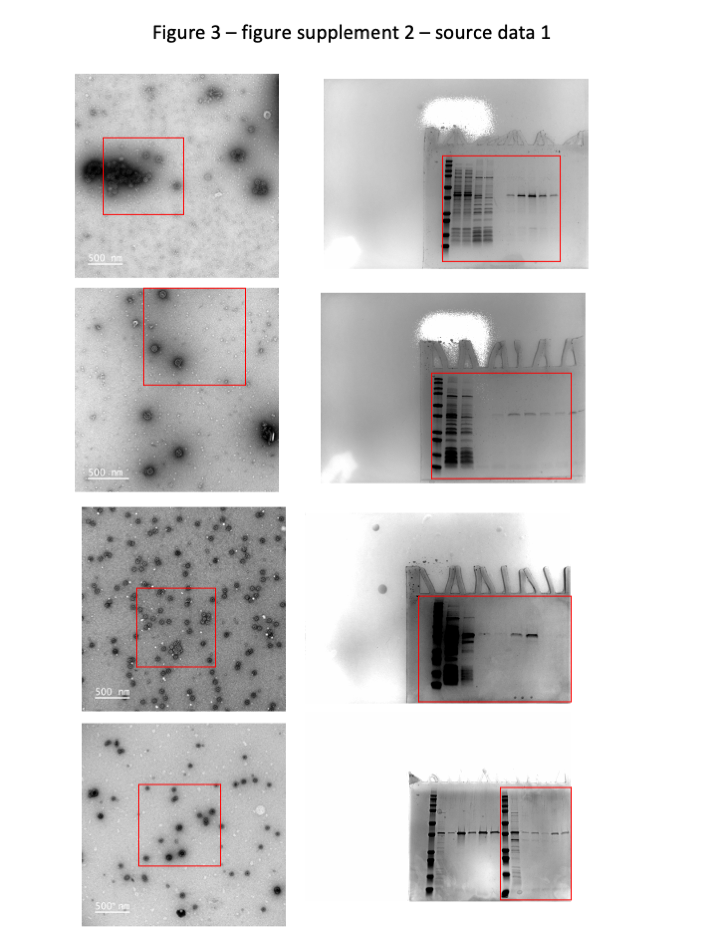

Supplement: Figure 3—figure supplement 2—source data 1. [file elife-87611-fig3-figsupp2-data1.zip › Figure_3_figure_supplement_2_source_data_1_crop.tiff]

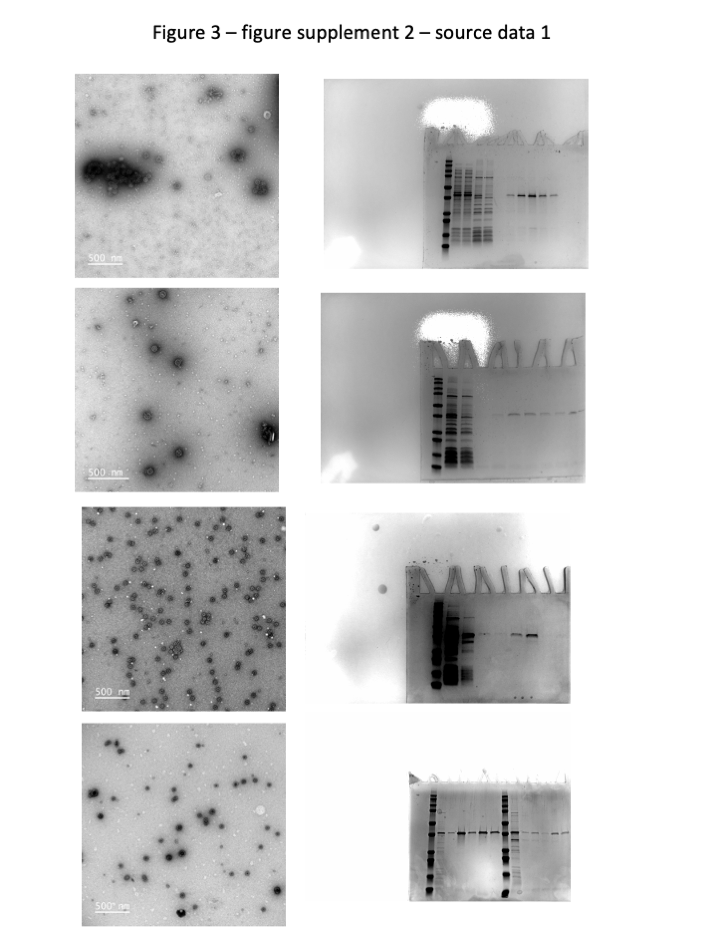

Supplement: Figure 3—figure supplement 2—source data 1. [file elife-87611-fig3-figsupp2-data1.zip › Figure_3_figure_supplement_2_source_data_1.tiff]

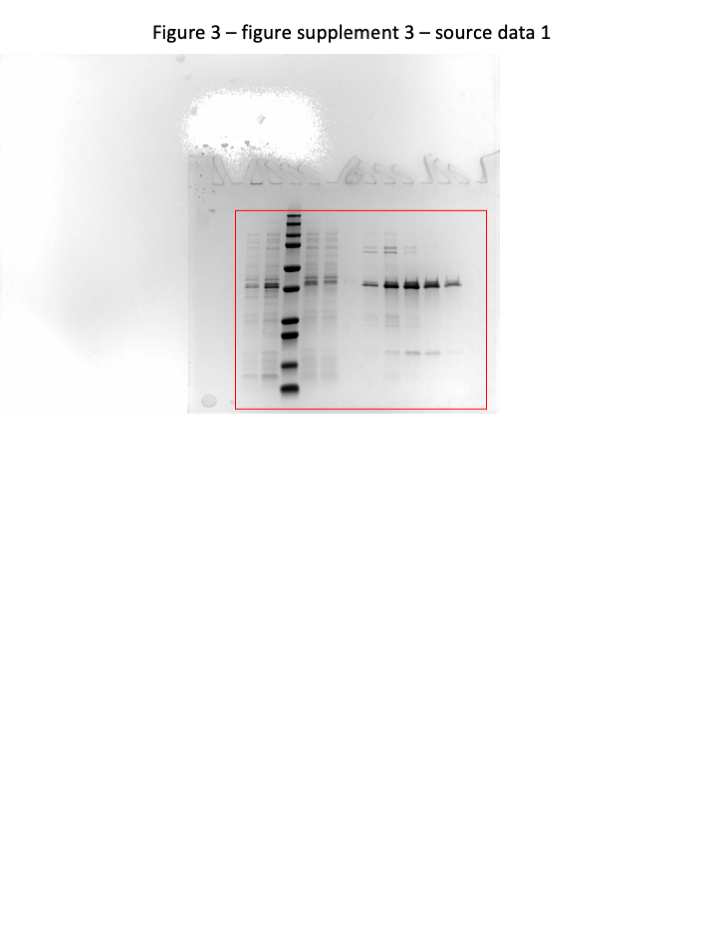

Supplement: Figure 3—figure supplement 3—source data 1. [file elife-87611-fig3-figsupp3-data1.zip › Figure_3_figure_supplement_3_source_data_1_crop.tiff]

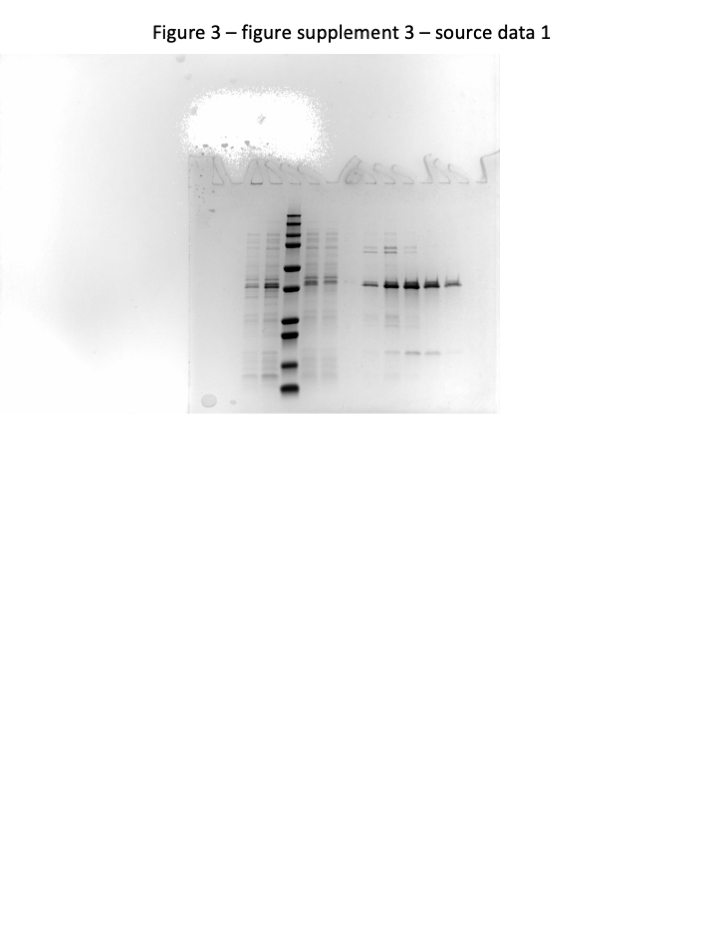

Supplement: Figure 3—figure supplement 3—source data 1. [file elife-87611-fig3-figsupp3-data1.zip › Figure_3_figure_supplement_3_source_data_1.tiff]

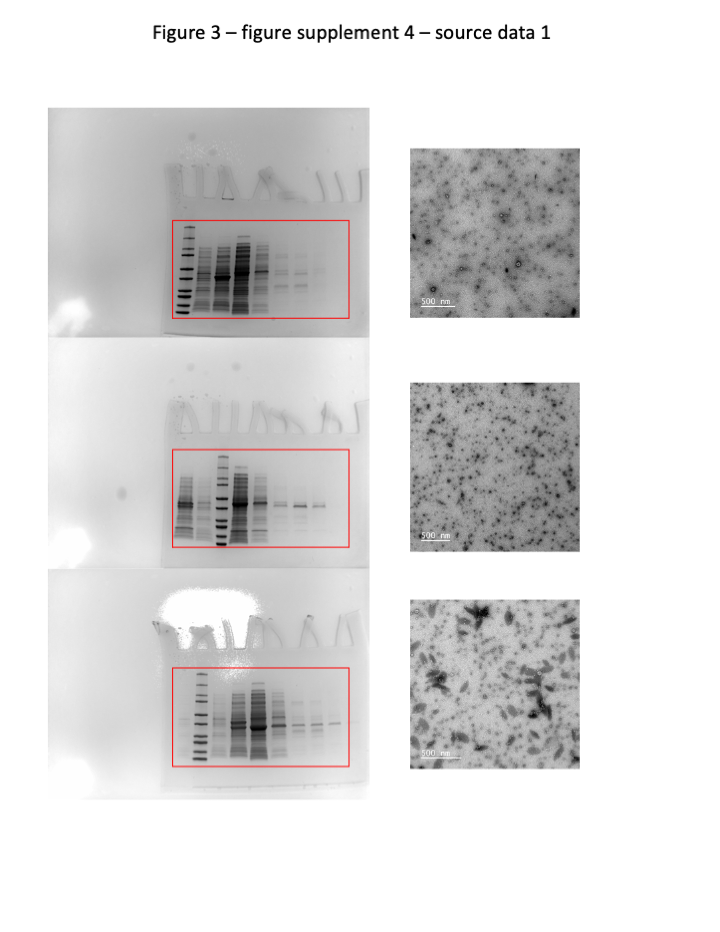

Supplement: Figure 3—figure supplement 4—source data 1. [file elife-87611-fig3-figsupp4-data1.zip › Figure_3_figure_supplement_4_source_data_1_crop.tiff]

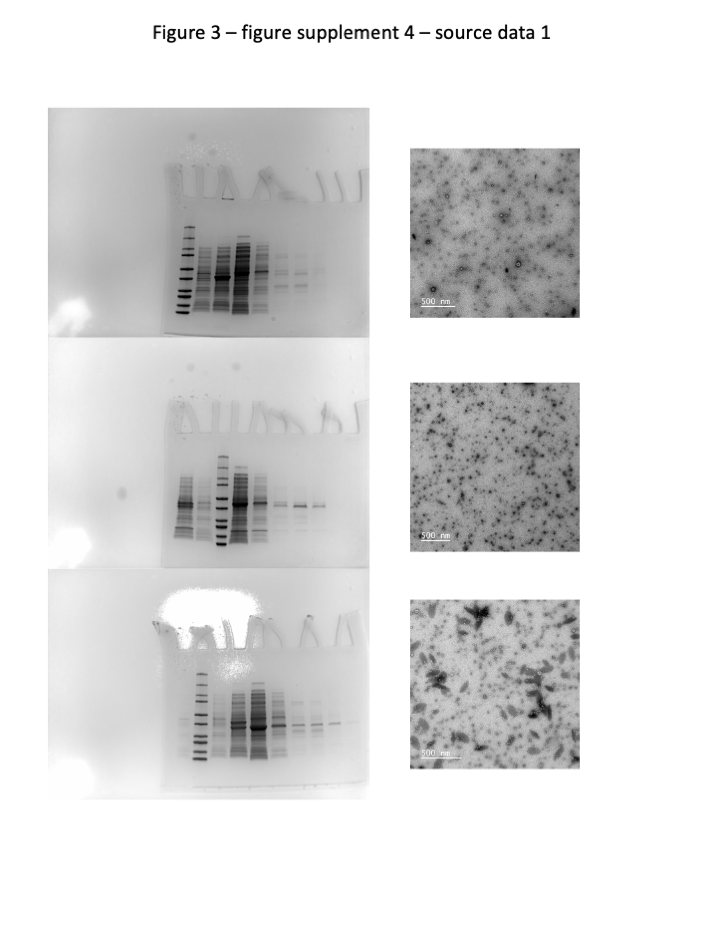

Supplement: Figure 3—figure supplement 4—source data 1. [file elife-87611-fig3-figsupp4-data1.zip › Figure_3_figure_supplement_4_source_data_1.tiff]

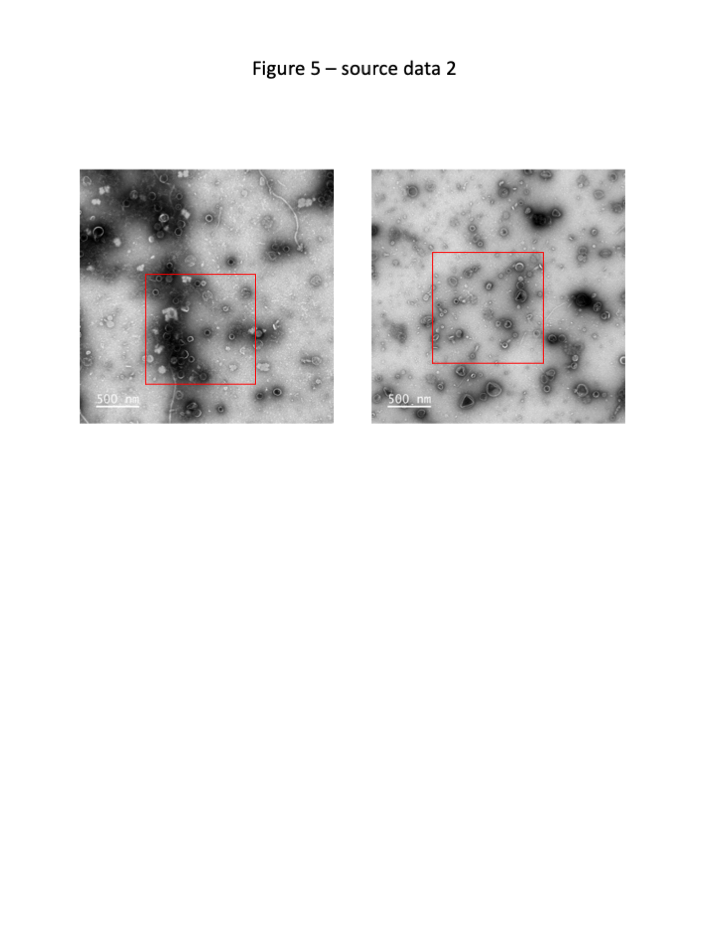

Supplement: Figure 5—source data 3. [file elife-87611-fig5-data3.zip › Figure_5_source_data_3_crop.tiff]

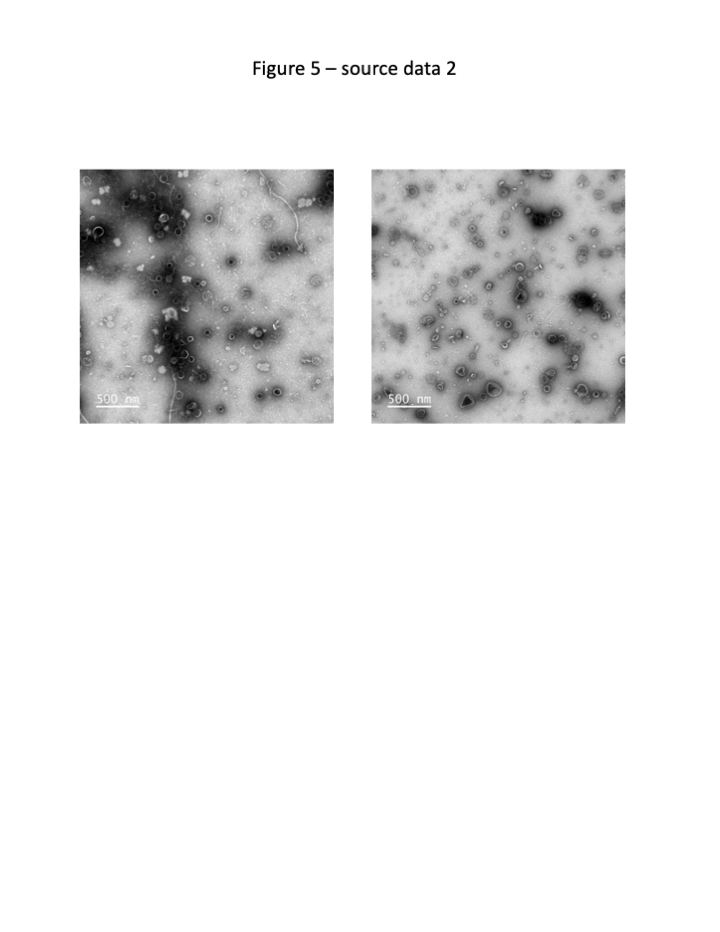

Supplement: Figure 5—source data 3. [file elife-87611-fig5-data3.zip › Figure_5_source_data_3.tiff]
